# Bayesian hierarchical negative binomial models for multivariable analyses with applications to human microbiome count data

**DOI:** 10.1371/journal.pone.0220961

**Published:** 2019-08-22

**Authors:** Amanda H. Pendegraft, Boyi Guo, Nengjun Yi

**Affiliations:** Department of Biostatistics, School of Public Health, University of Alabama at Birmingham, Birmingham, Alabama, United States of America; Wageningen Universiteit, NETHERLANDS

## Abstract

The analyses of large volumes of metagenomic data extracted from aggregate populations of microscopic organisms residing on and in the human body are advancing contemporary understandings of the integrated participation of microbes in human health and disease. Next generation sequencing technology facilitates said analyses in terms of diversity, community composition, and differential abundance by filtering and binning microbial 16S rRNA genes extracted from human tissues into operational taxonomic units. However, current statistical tools restrict study designs to investigations of limited numbers of host characteristics mediated by limited numbers of samples potentially yielding a loss of relevant information. This paper presents a Bayesian hierarchical negative binomial model as an efficient technique capable of compensating for multivariable sets including tens or hundreds of host characteristics as covariates further expanding analyses of human microbiome count data. Simulation studies reveal that the Bayesian hierarchical negative binomial model provides a desirable strategy by often outperforming three competing negative binomial model in terms of type I error while simultaneously maintaining consistent power. An application of the Bayesian hierarchical negative binomial model using subsets of the open data published by the American Gut Project demonstrates an ability to identify operational taxonomic units significantly differentiable among persons diagnosed by a medical professional with either inflammatory bowel disease or irritable bowel syndrome that are consistent with contemporary gastrointestinal literature.

## Introduction

The microbiome was first described as an “ecological community of commensal, symbiotic, and pathogenic microorganisms that literally share our body space” [1]. In more exact terminology, this definition is expressed as the totality of microbiota and associated genetic information interacting within an individual [2]. The current breadth of microbiome research is founded upon recent advances in next generation sequencing (NGS) technology. Namely, the accurate identification of microbes that constitute the microbiota and metagenome drives the potential to effectively understand the complex interactions and immense variability within and between the microbial colonies residing on and in the human body [3]. Of particular interest, NGS technology has supported large-scale endeavors such as the Human Microbiome Project [4] and MetaHIT [5], which collectively represent the aim to stimulate studies of the microbiome role in medical and public health settings. Significant relationships between microbes and disease have been identified, for example, in cases of inflammatory bowel disease (IBD) [6–8], diabetes [9–12], periodontal disease [13–15], and various cancers [16–19], among other medical conditions. Additionally, evidence has been presented to suggest significant changes associated with both demographic traits such as age [20–23] and race/ethnicity [20, 24, 25] and behavioral traits such as dietary habits [26–28] and antibiotic usage [26, 29–31]. A number of important questions concerning the commonalities and divergences in the microbiome, however, have yet to be addressed.

Microbial organisms are known to outnumber human cells by a ratio of at least ten to one with the majority of species present within the gastrointestinal tract [32]. One of the most commonly employed approaches to managing these extensive data is referred to as 16S rRNA gene amplicon sequencing. Through the utilization of a computational pipeline (e.g. QIIME, Mothur), amplified and sequenced hypervariable regions of the gene are filtered and binned into operational taxonomic units (OTUs) representing identifiable microbial taxa [3, 33–35]. Note that targeted amplicon sequencing does not reflect the sequence of the full genome, but, rather an inference of phylogeny by means of comparison to an existing 16S rRNA gene reference sequence database (e.g. GreenGenes, SILVA, Ribosomal Database Project) given a similarity threshold. The resulting human microbiome count data are then used to complete microbial diversity, community composition, and differential abundance analysis. Our primary interest involves the latter type of study. That is, our aim is to assess whether OTUs are significantly differentiable among subjects identified to have a disease or condition of interest in comparison to healthy controls by explicitly adjusting for dependencies between covariates (and also potential confounders, mediators, and moderators). More specifically, we employ the Bayesian hierarchical regression framework to complete differential abundance analyses of OTUs one-by-one by isolating relationships of interest while simultaneously controlling for multivariable sets of host characteristics as covariates.

Current literature shows that differential abundance analysis is complicated due to characteristics of microbiome count data such as over-dispersion and fluctuating library size. Fortunately, both of these challenges have been widely studied in the context of microarray and RNA-Seq experiments [36]. For example, analytical tools such as the R Bioconductor packages *edgeR* [37] and *DESeq2* [38, 39] have been developed so to adjust various library sizes by incorporating complex normalization techniques into the classical negative binomial (NB) model, which is known for handling over-dispersion. In contrast, software constructed in the context of microbiome count data such as the R Bioconductor package *metagenomeSeq* controls for similar challenges by transforming outcomes to relative abundance prior to modeling with a zero-inflated Gaussian mixture model [40]. These open-source packages are easily extended to a matrix-like format via wrapper functions of the R Bioconductor package *phyloseq* [41]. However, the assurance of an identifiable solution that is both precise and unbiased when utilizing said methods is severely limited by sample size. For example, *edgeR* and *DESeq* were developed with the small samples typical of RNA-Seq experiments in mind such that execution of the incorporated algorithms becomes overly time-consuming as the rows of a dataset increase. In the event that the number of host characteristics equals or exceeds the number of samples, methods of data reduction such as principal components or partial least squares incorporated into the multiple linear regression framework can offer a solution to this problem; yet, relevant information can be lost by selecting a pre-specified number of eigenvalue components or factors to accommodate the sample size. In order to address the complexities associated with modeling multivariable sets of tens or hundreds of host characteristics as covariates, we present a Bayesian hierarchical negative binomial (HNB) model capable of efficiently providing a comprehensive solution in respect to coefficient estimation without compromising type I error.

As we proceed, the Materials and Methods section will further describe the structure and challenges associated with human microbiome count data. Additionally, the parameterization and fitting of the proposed model will be specified followed by subsequent introduction of the American Gut Project (AGP) data. In the Results and Discussion section, we will discuss the results of extensive simulation studies in terms of performance criteria such as type I error, power, and false discovery rate. Application of the Bayesian HNB model in comparison to three competing NB models will be carried out on subsets of participants of the AGP including those individuals diagnosed by a medical professional with the gastrointestinal issues of inflammatory bowel disease and irritable bowel syndrome (IBS) compared to individuals deemed healthy. Through this application, we aim to highlight OTUs known to be significantly associated with the stated diseases while adjusting for numerous host characteristics such as dietary behaviors and systemic practices as covariates. The software necessary to carry out the proposed model is incorporated into the R package *BhGLM*, which can be freely downloaded at the GitHub repository, https://github.com/nyiuab/BhGLM.

## Materials and methods

### Attributes and challenges of human microbiome count data

Human microbiome count data is typically comprised of three basic components.

1. Counts, denoted as *c*_*ij*_, represent of the observed number of microbes for the *i*^*th*^ sample and *j*^*th*^ feature. For our purposes, a feature refers to an OTU or microbial taxon specified at any taxonomic level (e.g. species, genus, family).
2. Total reads, denoted as *T*_*i*_, are equivalent to the total number of counts observed for the *i*^*th*^ sample, i.e. $T_{i}=\sum_{j=1}^{m}c_{ij}$. This component is also referred to as library size.
3. Host characteristics, denoted as **X**_*i*_, represent clinical, physiological, environmental, behavioral, demographic, and/or genetic sample attributes. Note that *k* is used in the following text to stand for the multivariable set of tens or hundreds of host characteristics for a given sample, i.e. **X**_*i*_ = (*x*_*i*1_,*x*_*i*2_,…,*x*_*ik*_) where *p* = 1,2,…,*k*.

An example of the preceding components is found in Table 1, which highlights the goal to detect associations between features *c*_*ij*_ and host characteristics **X**_*i*_.

**Table 1:** Structure of human microbiome count data.

	Feature 1	Feature 2	⋯	Feature m	Total Reads	Host Characteristics
**Sample 1**	*c*_11_	*c*_12_	⋯	*c*_1*m*_	*T*_1_	**X**_1_
**Sample 2**	*c*_21_	*c*_22_	⋯	*c*_2*m*_	*T*_2_	**X**_2_
⋮	⋮	⋮	⋱	⋮	⋮	⋮
**Sample *n***	*c*_*n*1_	*c*_*n*2_	⋯	*c*_*nm*_	*T*_*n*_	**X**_*n*_

Human microbiome count data are subject to a number of challenges that require the development of adapted statistical tools. First, currently available NGS technologies do not have the ability to specify an exact number of sequences to be measured [34, 42]. This fluctuating library size yields OTU variability across samples that is not considered to be associated with any biological feature [43]. Hence, total reads should be accounted for prior to or as a part of statistical analysis via implementation of a normalization technique such as rarefying, scaling, or inclusion of a modeling offset to adjust a parametric generalized linear model (GLM). Note that the Bayesian HNB model employs the latter approach. A second challenge is that observed counts are over-dispersed, i.e. the variance of features is greater than the expected value. Therefore, standard Poisson models commonly used for analyzing count data are not appropriate, and models that have the ability to account for over-dispersion should be considered. Note that the Bayesian HNB model utilizes the NB distribution, which is known for handling over-dispersion to overcome this challenge. Lastly, due to modern capacities for data collection, human microbiome count data face the challenge in which the number of host characteristics can equals or exceeds the number of samples, and in many cases, the correlation among said multivariable host characteristics is complex.

### Negative binomial model

Similar to many existing methods, we aim to determine whether the abundance of a microbial taxon is statistically associated with host characteristics when testing features is completed one-by-one. For the simplicity of notation, we denote the count response for an analyzed microbial taxon as *y*_*i*_ = *c*_*ij*_. We assume the count response follows the NB distribution:

$$y_{i}∼NB(y_{i}|\mu_{i},\theta )=\frac{\Gamma (y_{i}+\theta )}{\Gamma (\theta )y_{i}!}\cdot {\left(\frac{\theta }{\mu_{i}+\theta }\right)}^{\theta }\cdot {\left(\frac{\mu_{i}}{\mu_{i}+\theta }\right)}^{y_{i}}$$ (1)

where *μ*_*i*_ and *θ* represent the mean and dispersion parameters, respectively, and Γ(∙) is the standard gamma function. It is well-known that *E*(*y*_*i*_) = *μ*_*i*_ and *Var*(*y*_*i*_) = *μ*_*i*_+*μ*_*i*_/*θ*. We can see that *Var*(*y*_*i*_)≥*E*(*y*_*i*_), and thus the NB distribution provides a way to deal with over-dispersion.

NB models relate the mean parameters *μ*_*i*_ to the predictors **X**_*i*_ via the link function logarithm:

$$\log\mu_{i}=X_{i}\beta$$ (2)

where **X**_*i*_*β* = *β*_0_+*x*_*i*1_*β*_1_+⋯+*x*_*ik*_*β*_*k*_. To account for the variablilty in library sizes among samples, we incorporate total reads *T*_*i*_ of each sample into the NB model by assuming that:

$$y_{i}∼NB(y_{i}|T_{i}r_{i},\theta )$$ (3)

$$\log r_{i}=X_{i}\beta$$ (4)

where *r*_*i*_ is the rate of the OTU of interest observed in the library of subject *i* and *T*_*i*_*r*_*i*_ is the mean parameter. Thus, the model is equivalent to:

$$y_{i}∼NB(y_{i}|\mu_{i},\theta )$$ (5)

$$\log(\mu_{i})=\log(T_{i})+X_{i}\beta$$ (6)

where log(*T*_*i*_) is the modeling offset correcting for the variability in library sizes.

### Hierarchical negative binomial model

The classical NB model described above cannot adjust for multivariable sets of tens or hundreds of host characteristics equaling or exceeding the number of samples. Thus, we propose a Bayesian HNB model in which the coefficients are themselves modeled, i.e. given prior distributions [44, 45]. Appropriate prior distributions can constrain the coefficients to lay within a reasonable range, which allows the Bayesian HNB model to handle many, highly correlated, covariates. We describe the Bayesian HNB model with commonly utilized Student’s *t* priors, although other priors can be used. The Student’s *t* distribution $t_{\nu }(0,s_{p}^{2})$ is expressed as a mixture of a Normal distribution in which unknown variances follows an inverse-*χ*^2^ distribution. That is,

$$\beta_{p}∼N\left(0,\tau_{p}^{2}\right),\;\mathrm{where}\;\tau_{p}^{2}∼\mathrm{inverse}∼\chi^{2}\left(\nu ,s_{p}^{2}\right)$$ (7)

where *ν*>0 and *s*_*p*_>0 denote the hyper-parameters for the degrees of freedom and scale, respectively [44, 45].

Note that *ν* and *s*_*p*_ are responsible for the amount of shrinkage imposed on the *β*_*p*_ regression coefficient estimates. We usually set *ν* to be the value of 1, which leads to a Cauchy prior. Then, smaller values of *s*_*p*_ induce stronger shrinkage forcing *β*_*p*_ closer to zero [46]. It is recommended that users consider the results of several prior scale values covering a reasonable range to ensure selection of an optimal model in terms of an adjusted Akaike’s information criteria (AIC) based on the effective number of parameters [47]. Moreover, *s*_*p*_ can be set differently for each covariate to induce varying amounts of shrinkage relative to biological importance. However, if no information is available, a common scale hyper-parameter *s* is acceptable for all variables.

### EM-IWLS algorithm for fitting the Bayesian HNB model

The Bayesian HNB model is fit by finding the posterior modes of the parameters, i.e. estimating the parameters by maximizing the posterior density. The log joint posterior distribution is derived as:

$$\begin{array}{l} \log\;p(\beta ,\theta ,\tau^{2}|y)=\log\;p(y|\beta ,\theta )+\sum_{p=0}^{k}\log\;p(\beta_{p}|\tau_{p}^{2})+\sum_{j=p}^{k}\log\;p(\tau_{p}^{2}|s_{p}^{2})\\ \;\propto \sum_{i=1}^{n}NB(y_{i}|\mu_{i},\theta )-\sum_{p=0}^{k}\left(\frac{\log\;\tau_{p}^{2}}{2}+\frac{\beta_{p}^{2}}{2\tau_{p}^{2}}\right)+\sum_{p=1}^{k}\left(\frac{\nu }{2}\log\;s_{p}^{2}-\frac{\nu +2}{2}\log\;\tau_{p}^{2}-\frac{\nu s_{p}^{2}}{2\tau_{p}^{2}}\right). \end{array}$$ (8)

An expectation-maximization (EM) algorithm incorporated into a modified iterative weighted least squares (IWLS) process is employed to fit the model as follows.

1. Initialize the model parameters (*β*,*θ*,*σ*) and unknown variances *τ*^2^ with plausible values.
2. For *t* = 1,2,…
  - E-step: Calculate the conditional expectation of (8) by updating $\tau_{p}^{-2}$ according to its conditional posterior expectation. For the Student’s *t* distribution, the conditional posterior distribution is written as a scaled inverse-*χ*^2^:
    $$\tau_{p}^{2}|\beta_{p},s_{p}^{2}∼\mathrm{Inv}‐\chi^{2}\left(1+\nu ,\frac{\nu s_{p}^{2}+\beta_{p}^{2}}{1+\nu }\right)$$ (9)
    yielding the conditional posterior expectation of
    $$E(\tau_{p}^{-2}|\beta_{p},s_{p}^{2})=\frac{1+\nu }{\nu s_{p}^{2}+\beta_{p}^{2}}.$$ (10)
    Note that only the conditional posterior expectation of $\tau_{p}^{-2}$ is necessary given its relationship to *β*_*p*_ through $\tau_{p}^{-2}$.
  - M-step: Based on *β*^*t*−1^ and *θ*^*t*−1^, approximate the pseudo-responses $z_{i}^{t}$ and pseudo-weights $w_{i}^{t}$ according to $NB(y_{i}|\mu_{i},\theta )\approx N(z_{i}|\eta_{i},w_{i}^{-1}\sigma^{2})$ in which *η*_*i*_ = log(*T*_*i*_)+**X**_*i*_*β*. Note that $z_{i}^{t}$ and $w_{i}^{t}$ are calculated according to the IWLS algorithm for fitting the classical NB model. Update *β* and *σ* by executing the hierarchical weighted normal regression,
    $$z_{i}^{\left(t\right)}∼N(\log(T_{i})+X_{i}\beta ,w_{i}^{-1(t)}\sigma^{2})\;\mathrm{where}\;\beta ∼N(0,\tau_{p}^{2})$$ (11)
    More specifically, the updated value of *β* is determined by deriving the conditional posterior mode that maximizes the expectation of the log conditional posterior distribution:
    $$\log\;p(\beta |y,\theta ,\tau^{2})\propto \sum_{i=1}^{n}\log N(z_{i}|\eta_{i},\;w_{i}^{-1}\sigma^{2})+\sum_{p=0}^{k}\log\;N(\beta_{p}|0,\tau_{p}^{2})$$ (12)
    for which $\tau_{p}^{2}$ is the value found via the E-step. Conditional on *β*, the dispersion parameter *θ* is updated by maximizing the NB likelihood $l(\theta )=\sum_{i=1}^{n}NB(y_{i}|\hat{\mu_{i}},\theta )$ using the Newton-Raphson algorithm.
3. Repeat the preceding step until convergence is achieved. That is, |*d*^(*t*)^−*d*^(*t*−1)^|/(0.1+|*d*^(*t*)^)<*ϵ* where *d*^(*t*)^ = −2*NB*(*y*|*μ*^(*t*)^,*θ*^(*t*)^) represents the *t*^th^ deviance estimate of the iteration and *ϵ* is a small value (e.g. 10^−5^).

Following the convergence of the EM-IWLS algorithm, hypothesis testing is possible through the maximum likelihood estimation of the coefficients, denoted as $\hat{\beta }$. These values are used calculate the test statistics $U_{p}={\hat{\beta }}_{p}/\surd Var({\hat{\beta }}_{p})$, which are known to approximately follow the standard Normal distribution. Thus, significance tests with null and alternative hypotheses, *H*_0_:*β*_*p*_ = 0 and *H*_*a*_:*β*_*p*_≠0, respectively, are available to return both p-values and confidence intervals at a pre-specified significance level.

A diagram of the Bayesian HNB model described above is provided in S1 Fig to aide interpretation of the relationships between hyper-parameters and parameters.

### Software availability

The proposed Bayesian HNB model is implemented using the function *bglm*, which is a part of the R package *BhGLM*. In addition to the Student’s *t* prior described above, the *bglm* function can also utilize three other prior distributions: double-exponential, spike-and-slab mixture Student’s *t*, and spike-and-slab mixture double-exponential. Again, *BhGLM* is freely available from the GitHub repository, https://github.com/nyiuab/BhGLM, which includes step-by-step guidelines for downloading *BhGLM* and implementing its functions.

### The American Gut Project

In order to demonstrate the usefulness of the proposed model, it was applied to subsets of the AGP as published to its publicly accessible repository on May 18, 2017 (ftp://ftp.microbio.me/AmericanGut/latest/11-packaged.zip). This link, organized by rarefaction depth and sequence trim length, contains numerous forms of the latest versions available for fecal, oral, and integumentary body sites. Our primary interest includes the 35580 OTUs observed across 12546 individuals provided for the fecal body site given unrarefied, untrimmed reads before binning. A total of 987 unique taxa at the species-level were observed for this dataset. These taxa belong to 1273 genera, 407 families, 309 orders, 164 classes, 65 phyla, and 2 kingdoms. Note that more genera were detected because a large number of taxa were not identifiable at the species-level. The minimum, lower quantile, median, upper quantile, and maximum of total reads across all individuals was found to be 1.00, 9290.75, 17420.00, 27475.75, and 517243.00, respectively. At this point, it is important to note that the AGP datasets are built upon polymerase chain reaction targeted gene amplicon sequencing of a 150 base pair segment of the 16S rRNA gene V4 region. OTU picking and taxonomic assignment were determined at a 97% species-level sequence identity clustered against Greengenes version 13.8 following application of the SortMeRNA 2.0 alignment tool. Note that a more complete description of microbiome samples and associated collection protocols are available at (https://github.com/biocore/American-Gut/blob/master/ipynb/).

Among the 204 host characteristics collected by the AGP self-administered questionnaire were history of IBD and IBS diagnoses. Accepted responses were comprised of “I do not have this condition,” “Self-diagnosed,” “Diagnosed by a medical professional (doctor, physician assistant),” and “Diagnosed by an alternative medicine practitioner”. In order to ensure validity, only those participants who reported “I do not have this condition” and “Diagnosed by a medical professional (doctor, physician assistant)” were considered for application. Samples were excluded if the criteria shown in Table 2 were not satisfied. The remaining samples resulted in a slightly elevated rate of IBD among AGP participants (2.96%) compared to the American adult population (1.3%) [48]. Alternatively, the rate of IBS among AGP subjects (5.21%) was slightly deflated below the expected range (5.70%-11.70%) among American adults dependent on diagnosis criteria [49]. These deviations are thought to be linked to motivation for participation. For example, persons diagnosed with some gastrointestinal conditions, IBD known to be more severe than IBS, are more likely to engage in human microbiome research [50, 51]. Hence, any generalizations of the proceeding results should be treated with utmost caution. Summary statistics for the AGP subsets following and prior to application of the criteria shown in Table 2 are found in S1 Table and S2 Table.

**Table 2:** Exclusion criteria for American Gut Project application.

Corrected age between 18 and 69
Country of residence reported as United States
No self-reported history of antibiotic usage in the past year
No self-reported history of cancer
No self-reported history of diabetes

## Results and discussion

### Simulation study design

Extensive simulation studies were completed to evaluate the performance of the proposed Bayesian HNB model to better understand its statistical properties and behaviors. As a means of comparison, the NB model was additionally considered in the form of three competing models. First, the classical NB model was implemented by the function *glm*.*nb* incorporated into the R package *MASS*. Then, two modified NB models implemented via edgeR and *DESeq2* were considered to reflect the broad utilization of these analytical tools in the analysis of human microbiome count data. In order to minimize any possible bias and yield reasonable responses similar to real human microbiome count data, a wide range of parameter values were considered.

Following the framework laid out in [52], human microbiome count data was simulated from the NB distribution defined in (1) for *n* = 50,100,200, and 500 samples and *k* = 1,50,100, and 200 covariates. That is, for **X** representative of a *n*×*k* simulated design matrix and *β* representative of a *k*×1 vector of simulated coefficient estimates, subject-specific systematic components were generated as:

$$\eta_{i}=\mu_{i}+X_{i}\beta =\left(\log\left(T_{i}\right)+\mu \right)+X_{i}\beta$$ (13)

such that

$$y_{i}∼NB(y_{i}|\exp\{\eta_{i}\},\theta )=\frac{\Gamma (y_{i}+\theta )}{\Gamma (\theta )y_{i}!}\cdot {\left(\frac{\theta }{\exp\{\eta_{i}\}+\theta }\right)}^{\theta }\cdot {\left(\frac{\exp\{\eta_{i}\}}{\exp\{\eta_{i}\}+\theta }\right)}^{y_{i}}$$ (14)

for *i* = 1,2,…,*n*. Note that this simulation was facilitated by the functions, *sim*.*x*, *sim*.*eta*, and *sim*.*y*, each implemented in *BhGLM*. The *n*×*k* simulated design matrix was populated using a random number generator for the multivariate normal distribution with a mean equal to 0 and a covariance matrix represented by ***V***. Specifically, ***V*** was designed to be the product of a single simulated correlation coefficient *ρ* and a *k*×*k* identity matrix. The correlation coefficient *ρ* was simulated for each set of human microbiome count data from the uniform distribution as either strong negative from range (−0.8,−0.5), weak from range (−0.1,0.1), or strong positive from range (0.5,0.8). With *T*_*i*_ and *μ* respectively representative of total reads and overall mean, the modeling offset log(*T*_*i*_) was ensured to fall in the range (7.1,10.5) by randomly sampling the scaling factor log(*T*_*i*_)+*μ* from the uniform distribution with range (0.1,3.5) and setting *μ* to the value of −7. The dispersion parameter *θ* was set to be uniformly sampled from the range (0.1,5.0) yielding moderate or large levels of over-dispersed counts. A total of nine prior scales, *s* = 0.01,0.05,0.10,0.15,0.25,0.50,0.75,1.00, and 2.00, were considered without allowance for varying covariate importance. For each combination of parameter values (i.e. 84 combinations), data simulation was iterated 100 times prior to application of the models (i.e. 12 models per iteration). The ranges of all simulated parameters are further summarized in Table 3.

**Table 3:** Summary of simulation study parameter ranges.

Parameter	Range
Sample Size, *n*	50, 100, 200, 500
Number of Coefficients, *k*	1, 50, 100, 200
Effect Size, *β*_*p*_	Zero: 0 Non-zero small: Uniform(0.01,0.15) Non-zero moderate: Uniform(0.20,0.35) Non-zero large: Uniform(0.40,0.55)
Modeling Offset, log(*T*_*i*_)	Uniform(7.1,10.5)
Dispersion, *θ*	Uniform(0.1,5)
Correlation, *ρ*	Strong negative: Uniform(−0.8, −0.5) Weak: Uniform(−0.1,0.1) Strong Positive: Uniform(0.5,0.8)
Prior Scale, *s*	0.01, 0.05, 0.10, 0.15, 0.25, 0.50, 0.75, 1.00, 2.00

Under the hypotheses of *H*_0_:*β*_*p*_ = 0 versus *H*_*a*_:*β*_*p*_≠0, simultaneous significance testing of coefficient estimates was completed based on a significance level of 0.05. In the setting of *k* = 1, four sets of simulations were executed with a single coefficient estimate equated to the value of 0 or a non-zero effect size uniformly drawn from small range (0.01,0.15), moderate range (0.20,0.35), or large range (0.40,0.55). Moreover, in the settings of *k* = 50,100, and 200, a single coefficient estimate was assigned to have a non-zero effect uniformly sampled from each of the stated ranges to yield analogous comparisons. In the proceeding discussion, arbitrarily selected covariates labeled with *β*_15_, *β*_30_, and *β*_45_ represent the three non-zero effect sizes, respectively. All additional effect sizes were set to be the value of 0.

Type I error and power were calculated for zero and non-zero coefficient estimates one-by-one. Then, aggregate performance was evaluated using false discovery rate (FDR), measured as the expectation of false discovery proportion (FDP) relative to nominal thresholds, and receiver operating characteristic (ROC) curves, measured as true positive rate (TPR) plotted against false positive rate (FPR) under different p-value thresholds. The accuracy of coefficient and over-dispersion estimation was assessed according to mean deviations. The numeric and graphic descriptions of the described simulation studies depended upon R version 3.5.0 and the high-performance computing resources of the University of Alabama at Birmingham Cheaha cluster and the following R packages: *reshape*, *ggplot2*, *colorspace*, *grid*, and *gridExtra*. The reproducible simulation code used to generate the whole simulation data and to complete these studies is available at https://github.com/ahpendegraft/HNB.

### Simulation study results

**Fig 1:**
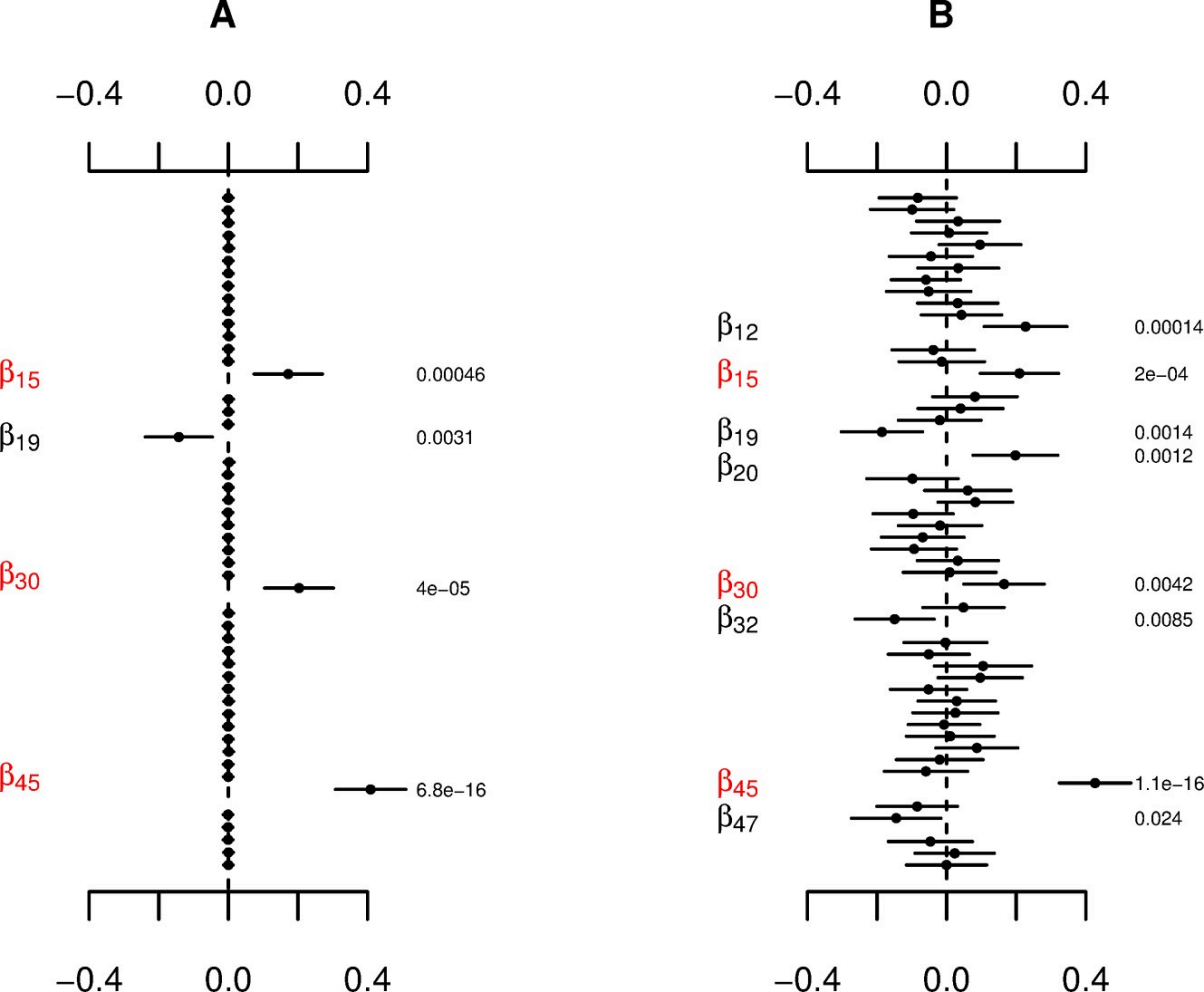
Comparison of the Bayesian HNB and classical NB models in respect to a single simulation of human microbiome count data. Jointly fitting 200 samples in relationship to 50 covariates based on a design matrix simulated using weak correlation coefficient, *ρ*∈(−0.10,0.10), with (A) the Bayesian HNB model and (B) the classical NB model. All nine prior scales were considered for the fitting of the Bayesian HNB model with *s* = 0.01 selected based on a minimized adjusted AIC of 1149.94. The three simulated non-zero effects, *β*_15_∈(0.01,0.15), *β*_30_∈(0.20,0.35), and *β*_45_∈(0.40,0.55), are displayed in red. The points, spanning lines, and right-hand side numbers represent coefficient estimates, ±2 standard errors, and p-values, respectively.

Fig 1 displays coefficient estimates, standard errors, and p-values for a comparative application of the Bayesian HNB and classical NB models on a single simulation of human microbiome count data. The results from the two models are markedly different. Both approaches were capable of identifying the three non-zero effects; however, the classical NB model yielded more false positives as shown in Fig 1B. This stand-alone example precedes our expectation that the Bayesian HNB model outperforms the classical NB model in terms of type I error while maintaining consistent power.

Prior to our assessment of multivariable sets involving tens or hundreds of covariates, it is important to assess type I error and power in the setting of human microbiome count data with *k* = 1. No significant difference in either of the said statistical properties appears to be subject to selection of the Bayesian HNB model over the three competing models. Type I error ranges from 0.01 to 0.04 between the prior scales of *s* = 0.01 to *s* = 2.00 for the Bayesian HNB model and is observed to be 0.05, 0.04, and 0.09 for *MASS*, *edgeR*, and *DESeq2*, respectively. As anticipated, increases in the sample size, in general, decrease type 1 error and increase power as shown in S2 Fig. Moreover, the effect size is noticeably influential on power given on average 25%, 80%, and 95% of small, moderate, and large simulated non-zero effects are detected, respectively. Though the classical NB model maximizes said estimations of power estimated to be 70% on average, the Bayesian HNB model performs consistently with results indistinguishable for prior scales of *s* = 0.50 to *s* = 2.00 with ranging from 69% to 70%. *DESeq2* provides noticeably reduced power estimated to be 58% on average.

**Fig 2:**
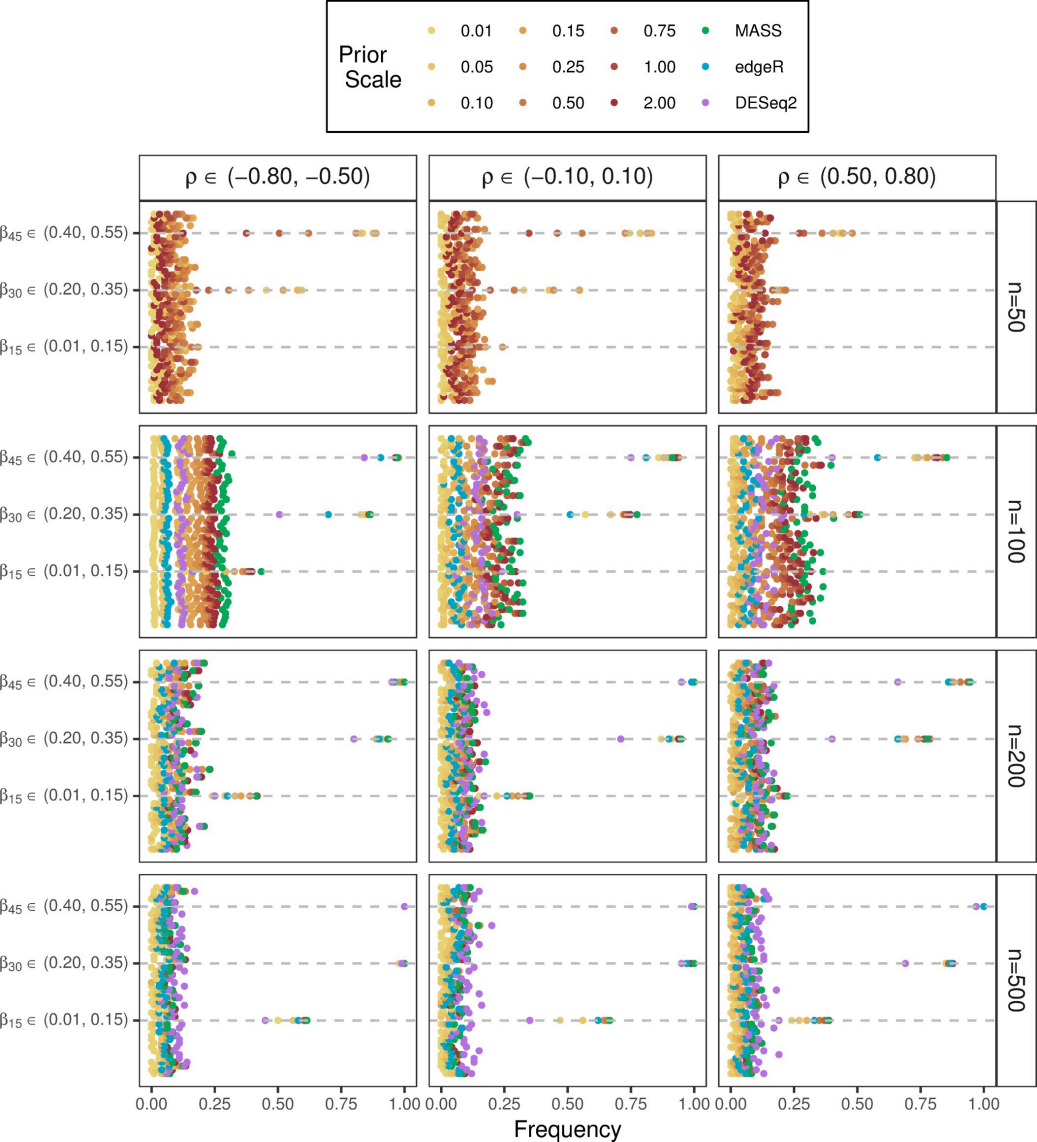
Comparison of the Bayesian HNB model and three competing models taking into account the simulation of human microbiome count data with 50 covariates. Jointly fitting 50 covariates using the Bayesian HNB model (yellows, oranges, and reds) compared to *MASS* (green), *edgeR* (blue), and *DESeq2* (purple) stratified by sample size (right) and correlation (top). Increasing prior scale is represented by an increasing sequential palette detailed in the legend. Each row of the individual panels represents one of the 50 covariates with the gray dashed lines associated with *β*_15_∈(0.01,0.15), *β*_30_∈(0.20,0.35), and *β*_45_∈(0.40,0.55) corresponding to the three simulated covariates assigned to have non-zero effects from small, moderate, and large range, respectively. Frequency along said gray dashed lines represents power. All additional effects were simulated to be the value of 0 such that frequency represents type I error.

Fig 2 presents a comparison of the Bayesian HNB model and three competing models by taking into account the simulation of human microbiome count data with 50 covariates. The term frequency, shown on the x-axis, corresponds to type I error for simulated zero effects and power for simulated non-zero effects. More specifically, each row of the depicted panels provides information about an individual coefficient estimate such that the stated measures are computed according to univariate formulations. Only the joint fitting of 50 covariates is graphically provided; additional results involving larger multivariate sets of 100 and 200 covariates are shown in S3 Fig and S4 Fig, respectively.

Type I error and power for the proposed Bayesian HNB model considering coefficient estimates one-by-one are both significantly influenced by prior scale and sample size. For example, type I error increases from 1% to 5% and power decreases from 33% to 8% across increases in prior scale from *s* = 0.01 to *s* = 2.00 for the Bayesian HNB model when sample size and covariates are held constant at *n* = 50 and *k* = 50, respectively. At this point, it is important to reiterate the classical NB model is non-identifiable in cases of *n*≤*k* explaining the absence of blue points of along the first row of panels in Fig 2. In contrast, type I error increases from 1% to 12% and power increases from 64% to 73% across increases in prior scale from *s* = 0.01 to *s* = 2.00 for the Bayesian HNB model for the larger sample size of *n* = 200 and *k* = 50 fixed. The classical NB model for said parameters results in a type I error estimation of 12% and a power estimation of 76%. Moreover, *edgeR* and *DESeq2* yield type I error estimates of 6% and 11% and power estimates of 66% and 55%, respectively. These comparisons support the hypothesis that the proposed Bayesian HNB model provides a viable alternative model in situations of multivariable sets of host characteristics Given larger sample sizes such as *n* = 500, type 1 error rate fluctuates between 1% and 7% on average across increases in prior scale from *s* = 0.01 to *s* = 2.00 for the proposed model; a performance that is at or markedly below the *MASS* and *DESeq2* type I error rate estimations of 7% and 10%, respectively. Note that *edgeR* performs comparably for *n* = 500 given a type I error rate estimation of 5%. Power is additionally affected by the correlation coefficient and effect sizes imposed on the simulated covariates. That is, for strong negative, weak, and strong positive settings, power to detect small non-zero effects is observed to be approximately 59%, 62%, and 33% on average when sample size is held constant at *n* = 500 with minimal fluctuations across increases in prior scales. The competing models result in a similar trend with power observed to be approximately for the stated parameter settings with a minimized power provided by *DESeq2* observed for the strong positive setting. In contrast, power to detect moderate and large non-zero effects for the same correlation coefficients is greater than the nominal level of 80% on average for both the Bayesian HNB model and three competing models given a sample size greater than or equal to *n* = 200.

**Fig 3:**
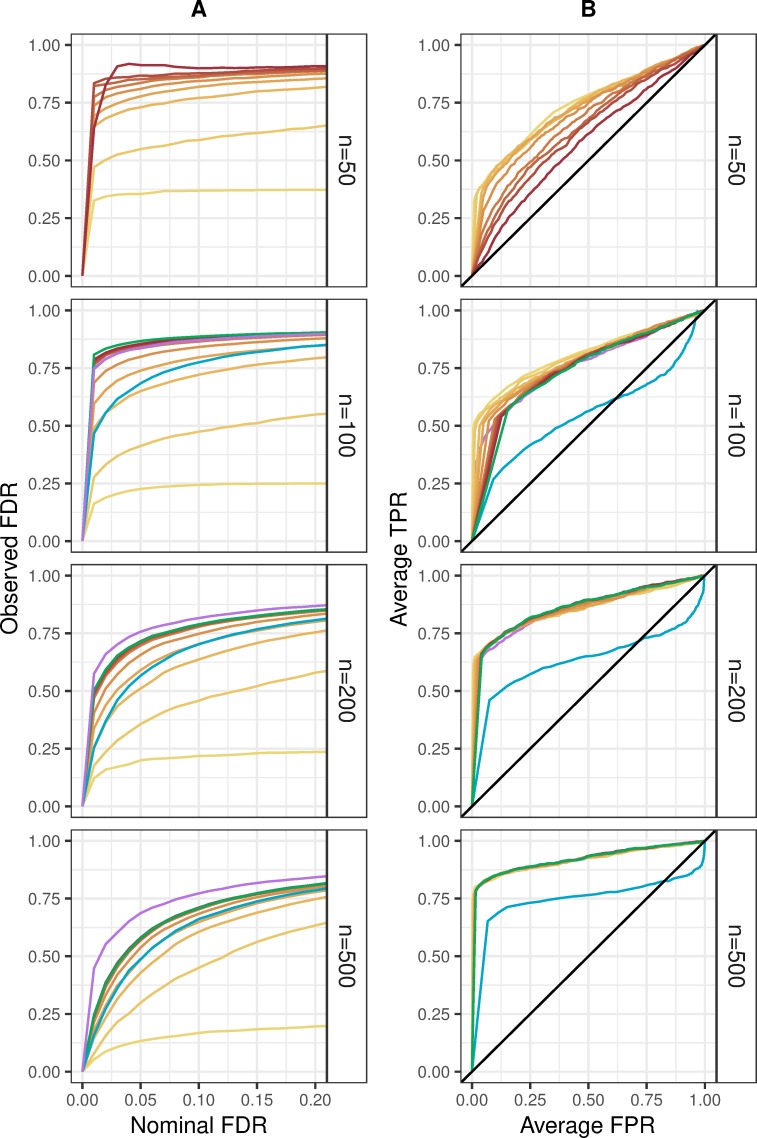
Performance criteria for the simulation of human microbiome count data with 50 covariates. (A) FDR control, shown as the expectation of FDP against nominal thresholds, and (B) power comparisons, shown as ROC curves of TPR against FPR under different p-value thresholds, for jointly fitting 50 covariates using the Bayesian HNB model (yellows, oranges, and reds) compared to *MASS* (green), *edgeR* (blue), and *DESeq2* (purple) stratified by sample size (right). Increasing prior scale is represented by an increasing sequential palette detailed in the legend. Each iteration included three covariates simulated to have non-zero effect sizes defined as *β*_15_∈(0.01,0.15), *β*_30_∈(0.20,0.35), and *β*_45_∈(0.40,0.55).

The ability to control FDR was assessed for the Bayesian HNB model compared to the classical NB model. As shown in Fig 3A, FDR was markedly elevated for the Bayesian HNB model with weak shrinkage ranging between *s* = 0.10 and *s* = 2.00 as well as for classical and modified NB models relative to nominal significance levels between 0.00 and 0.20. Looking more closely, FDR control worsened for said models at higher nominal values. In contrast, the Bayesian HNB model with strong shrinkage ranging between *s* = 0.01 and *s* = 0.10 achieved better FDR control, though slight FDR inflation was observed for all approaches. Next, power was compared using ROC curves. Fig 3B displays average TPR for the Bayesian HNB model to be higher than average TPR for the classical NB model given average FPR fixed at or below 0.15, 0.04, and 0.02 for sample sizes of *n* = 100, *n* = 200, and *n* = 500, respectively, regardless of prior scale. Moreover, the Bayesian HNB model showed significantly larger area under the curve than *edgeR* while *MASS* and *DESeq2* performed on par for sample size greater than or equal to *n* = 200. In light of these aggregate performance criteria, the Bayesian HNB model with prior scale between *s* = 0.01 and *s* = 0.10 yielded the best FDR control without compromising power.

**Fig 4:**
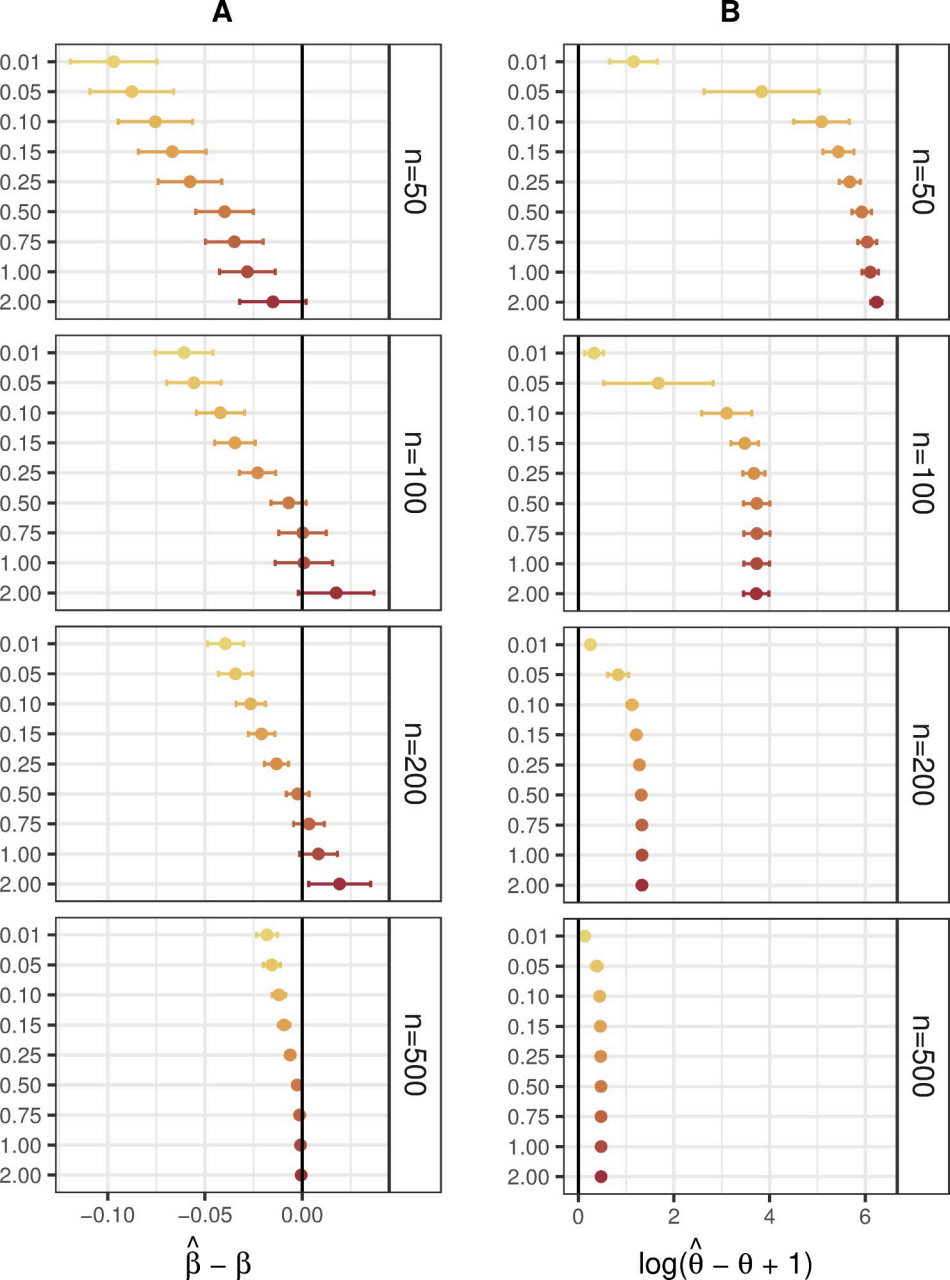
Accuracy of coefficient and overdispersion estimation for the simulation of human microbiome count data with 50 covariates. Deviations between estimated and simulated (A) coefficients and (B) over-dispersions for jointly fitting 50 covariates using the Bayesian HNB model (yellows, oranges, and reds) stratified by sample size (right). Increasing prior scale is represented by an increasing sequential palette detailed in the legend. The points and spanning lines represent means and ±2 standard errors, respectively.

The accuracy of coefficient and over-dispersion estimation for the proposed Bayesian HNB model in comparison to the classical NB model is presented in Fig 4. It is notable that estimates of *β* are close to the corresponding simulated values given all parameter settings with mean differences maintained between -0.28 and 0.22 on average. These mean deviations are largest for the prior scales of *s* = 0.01 and *s* = 0.05 when the number of covariates is set to equal or exceed the sample size for the Bayesian HNB model. Moreover, increases in sample size from *n* = 50 to *n* = 500 and decreases in the number of covariates from *q* = 200 to *q* = 1 reduce deviations to be values nearly indistinguishable from the value of 0; that is decreases in differences on average drop from -0.06 to -0.01 and from -0.03 to -0.02, respectively. Increases in effect size induce underestimated coefficients, whereas the simulated correlation coefficient provided no obvious effect. Opposite trends are observed for the accuracy of dispersion estimation. That is, estimates of *θ* incurred larger mean differences from the simulated values for larger scaling factors reaching a maximum of 13.55 when considered on the log plus 1 scale. These results support that the proposed Bayesian HNB model is capable of yielding an accurate fit even while robustly dealing with vast over-estimations of the over-dispersion parameter provided by classical and modified versions of the NB GLM.

In summary, our extensive simulation studies indicate that the Bayesian HNB model consistently results in better performance than the classical NB model implemented in *MASS* and the modified NB model implemented in *DESEq2* when considering type I error and FDR; *edgeR* showed provided similarly conservative estimates of said performance criteria However, this statistical property potentially comes at the cost of reduced power for the results of *BhGLM* (and *edgeR)* given patterned under-estimation of coefficients in parameter settings where prior scale and sample size are small as shown in Fig 4A. This trade-off must be carefully weighed according to scientific hypotheses of interest. In cases of multivariable sets of tens or hundreds of host characteristics exceeding sample size, the proposed method is often best given its ability to efficiently converge to an acceptably accurate model fit by shrinking the estimates of unimportant parameters with an optimal prior scale. More detailed information regarding computation time and convergence are available in S5 Fig and S6 Fig.

### Application to the American Gut Project

Differential abundance analysis was performed using the Bayesian HNB and classical NB models in order to determine whether the AGP data provide evidence to suggest OTUs are significantly differentiable between persons diagnosed by a medical professional with the gastrointestinal issues of IBD and IBS compared to healthy controls. These models allowed for the explicit adjustment for dependencies between covariates (and also potential confounders, mediators, and moderators) such as dietary behaviors and systemic practices. In order to replicate the scenario in which the number of host characteristics equals or exceeds the sample size, propensity score matching was utilized as implemented by the function *matchit* incorporated into the R package *MatchIt* version 3.0.2. Specifically, logistic regression was employed to estimate distance measures prior to nearest neighbor matching based on a one to one ratio. No subjects were discarded before matching based on the host characteristics of age (in years), body mass index, race (Caucasian, African American, all other races), and gender (male, female). The sample sizes for the differential abundance analysis of IBD and IBS were reduced by this technique to 76 and 126, respectively. Summary statistics for the reduced AGP subsets are found in S1 Table. Note that we excluded *edgeR* and *DESeq2* from these applications given similar results to *BhGLM* and *MASS*, respectively, as pointed out in the preceding discussion of simulation studies.

A multivariable set of 38 host characteristics operationalizing 32 dietary behaviors and 6 systemic practices were utilized as covariates for the differential abundance analysis of reduced AGP subsets. All said covariates were converted into indicator variables yielding 117 coefficients to be estimated beyond the intercept. Moreover, 104 missing values were imputed by corresponding sample means of the data prior to modeling. All nine prior scales, *s* = 0.01,0.05,0.10,0.15,0.25,0.50,0.75,1.00, and 2.00, were considered for the Bayesian HNB model with the value producing minimized adjusted AIC selected as optimal (S3 Table). All p-values were adjusted for multiple comparisons using the Benjamini-Hochberg procedure. The reproducible application code used to complete these analyses is available at https://github.com/ahpendegraft/HNB.

Alternative applications of the Bayesian HNB model were performed using full AGP subsets to be compared to the results provided by *MASS*. Age, body mass index, race, and gender were additionally included in the multivariable set of host characteristics for the differential abundance analysis of full AGP subsets increasing the number of coefficients to be estimated beyond the intercept to 121. Summary statistics for the full AGP subsets are found in S2 Table; adjusted AIC statistics and OTUs determined to be significantly differentially abundant for IBD and IBS are found in S3 Table and S4 Table, respectively.

**Fig 5:**
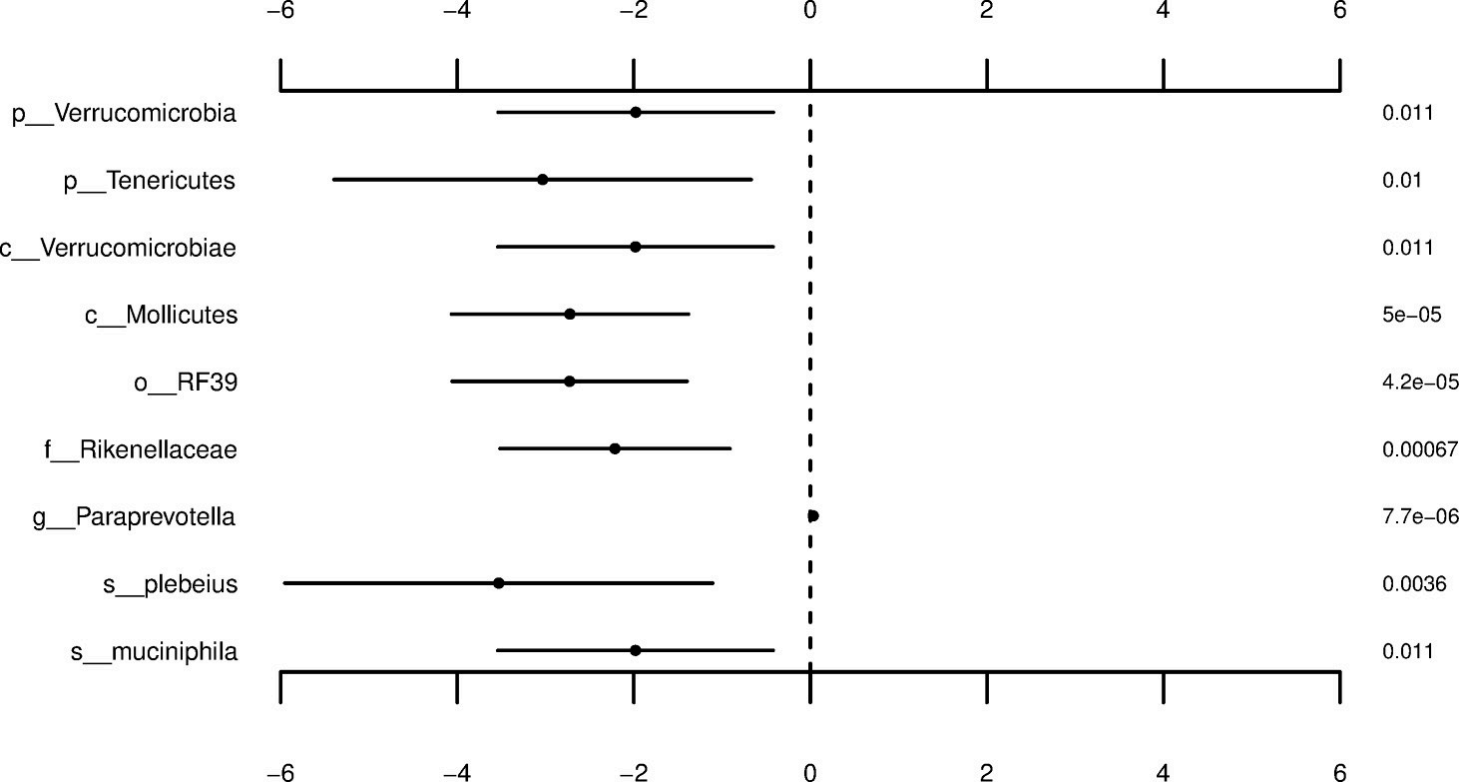
OTUs determined to be significant based on the differential abundance analysis of IBD utilizing the reduced AGP subsets. Coefficient estimates, ±2 standard errors, and p-values for differentially abundant taxa between participants specifying to have been diagnosed with IBD by a medical professional compared to participants specifying to have not been diagnosed with IBD by a medical professional. Statistical significance judged according to a significance level of 0.10 following the Benjamini-Hochberg correction for multiple comparisons. Taxonomic level and OTU name (left); coefficient estimate ±2 standard errors (middle); p-value (right).

After excluding taxa with a mean relative abundance < 0.1%, 20 species, 49 genera, 37 families, 21 orders, 15 classes, and 8 phyla remained for differential abundance analysis of IBD. Fig 5 shows the resultant microbial taxa identified to be significantly differentially abundant based on a decision rule of 0.10 with corresponding coefficient estimates, standard errors, and p-values. Of particular interest are the family-level taxon, Rikenellaceae, and the genus-level taxon, *Paraprevotella*, which present effects with opposing directions. Namely, Rikenellaceae was found to have a large negative coefficient estimate of -2.2138 and *Paraprevotella* was found to have a small positive coefficient estimate of 0.0302. These findings are consistent with current literature which reports the said microbiota are depleted and enriched in the colonic mucosa of IBD patients, respectively, particularly those diagnosed with Crohn’s disease [53, 54]. Other significantly differentially abundant OTUs included the species-level taxon, *B*. *plebeius*. This microbe,found to have a large negative coefficient estimate of -3.5289, conflicts with current literature, which suggests decreases in this OTU among patients in remission following ileocolonic resection for Crohn’s disease [55]. It is worthwhile to mention that insufficient evidence was provided to conclude a significant difference for the previously noted microbe *F*. *prausnitzii*. Also, OTUs at the species, genus, and family-level occupying large proportions of bacterial compositions failed to produced significant signals implying that the microbes shown in Fig 5 make up only a small portion of the total number of OTUs observed within the AGP subset focused on analysis of IBD. These results emphasize the importance of completing differential abundance analysis on features beyond those which comprise large proportions of observations and difficulty of identifying consistent signals warranting further development of efficient microbiome data analysis techniques [54].

**Fig 6:**
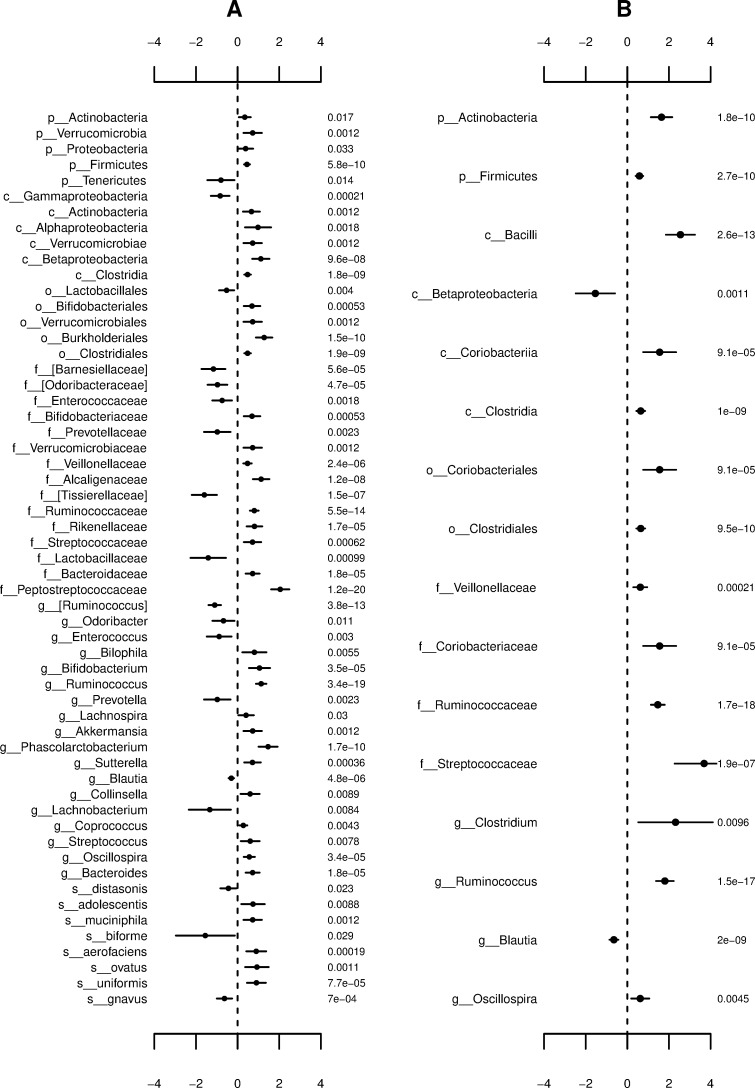
OTUs determined to be significant based on the differential abundance analysis of IBS utilizing the reduced AGP subsets. Coefficient estimates, ±2 standard errors, and p-values for differentially abundant microbial taxa between participants specifying to have been diagnosed with IBS by a medical professional compared to participants specifying to have not been diagnosed with IBS by a medical professional. Statistical significance judged according to a significance level of 0.10 following the Benjamini-Hochberg correction for multiple comparisons. Moreover, results represent **(A)** the Bayesian HNB model using optimal prior scale and **(B)** the classical NB model. Taxonomic level and OTU name (left); coefficient estimate ±2 standard errors (middle); p-value (right).

Taxa were again filtered according to a mean relative abundance < 0.1% before the differential abundance analysis focused on the samples allocated to the second question shown in S1 Table. This resulted in the consideration of 18 species, 37 genera, 30 families, 17 orders, 13 classes, and 7 phyla. Those microbial taxa identified to be significantly differentially abundant based on a decision rule of 0.10 are shown in Fig 6 with corresponding coefficient estimates, standard errors, and p-values. Notably, the majority of differences between the diseased and non-diseased groups was attributed to the OTUs nested within the phylum-level taxon, Actinobacteria, which itself was found to be enriched when all other coefficient estimates are held constant. More specifically, sufficient evidence was provided to conclude that those persons who self-reported to have been diagnosed with IBS by a medical professional saw significantly increased values of the log of expected counts for the genus-level taxa, *Collinsella*, *Akkermansia*, and *Bifidobacterium*, compared to persons who self-reported to have not been diagnosed with IBS by a medical professional. The coefficient estimates in this case were computed to be 0.6010, 0.7231, and 1.0526, respectively. These results are consistent with contemporary literature, which supports that these bacteria play a role in the mediation of IBS [56–58]. The microbe *C*. *aerofaciens* was found to have a large positive coefficient estimate of 0.8964 supporting its established association with the digestive symptoms of IBS and the presence of colorectal carcinoma tissue [17, 57]. The microbe *A*. *mucinophila* was found to have a large positive coefficient estimate of 0.7231 supporting its relationship with degradation of the mucus layer covering the gastrointestinal tract [7]. And, lastly, the microbe *B*. *adolescentis* was found to have a large positive coefficient estimate of 0.7410 supporting its usage as a chemical treatment for colitis [54]. Another significantly differentially abundant OTU worth mentioning is the genus-level taxon, *Prevotella*. Though consistent signals linking these microbes to IBS have yet to be presented, relationships with dietary behaviors such as increased consumption of red meat and whole grain carbohydrates are established [20, 28].

The numbers of overlapping significant associations found by each method using reduced and full AGP subsets are presented in S7 Fig. We emphasize the Bayesian HNB model applied to reduced AGP subsets was able to identify 9 and 57 OTUs significantly associated with IBD and IBS, respectively. In contrast, the classical NB model was non-convergent for the differential abundance analysis of IBD using the reduced AGP subsets given the numbers of coefficients to be estimated exceeded the number of samples). For the differential abundance analysis of IBS using the reduced AGP subsets, the classical NB model found 15 OTUs to be significant. Of these 15 OTUs, 11 were identified by the Bayesian HNB model.

We restate that it is necessary to interpret these results with utmost caution. This follows a number of limiting characteristics innate to the AGP such as self-selection for participation, potentially motivated by extreme dietary tendencies or painful effects of gastrointestinal issues, and self-administration for data collection utilized to sample both the microbiome and associated host characteristics, just to name a few. In light of these potential biases, it is possible to design a number of future microbiome analyses based on these data given rigorous development of exclusion criteria and scientific questions. Consistency with current literature supports that the proposed Bayesian HNB model is a useful tool for the detection of differentially abundant features which should be considered as means for downstream analysis for these projected studies.

## Conclusions

Previously developed methods designed for the statistical analysis of microbiome count data when considering OTUs one-by-one have heavily depended upon the incorporation of complex normalization techniques such as rarefying and scaling into NB models (e.g. *MASS*, *edgeR*, *DESeq)* or transformation of outcomes to relative abundance prior to utilization of the zero-inflated Gaussian mixture model (e.g. *metagenomeSeq*). The Bayesian HNB model avoids these steps allowing for the direct modeling of raw counts through the incorporation of library sizes as a modeling offset. More specifically, we have shown the proposed method is capable of simultaneously adjusting for multivariable sets of tens or hundreds of clinical, physiological, environmental, behavioral, demographic, and/or genetic sample host characteristics, which is not always attainable by the classical NB model implemented in *MASS* or modified NB models implemented in *edgeR* or *DESeq2* when the number of samples is restricted. This capacity yields the Bayesian HNB method a desirable strategy, particularly in the context of the large volumes of human microbiome data collectable by modern research practices. As verified by our extensive simulation studies, the proposed method provides an advantageous control over type I error by minimizing the number of false positives with a stringent prior scale hyper-parameter. However, said conservatism must be weighed in light of the scientific hypotheses of interest to ensure that the power necessary to detect all meaningful OTUs is available. Moreover, the analysis of AGP subsets highlighted that the selection of the prior scale hyper-parameter should be carefully considered over a range of reasonable values so to ensure the selection of an optimal model. As illustrated in the Application to the American Gut Project section, we recommend the use of the adjusted AIC statistic proposed by [47] to avoid the introduction of user bias. That is, degrees of freedom are adjusted to the effective number of parameters prior to calculation of the classical AIC statistic for each application of the Bayesian HNB model with minimized values providing stronger evidence for model selection. Moreover, real data analysis resulted in the identification of a number of microbes significantly differentially abundant in the guts of persons diagnosed by a medical professional with IBD and IBS compared to those persons self-reported to be unaffected. As a final remark, we mention that the Bayesian HNB model is applicable for other types of count data including RNA-Seq experiments which broadens the strength of this analytical tool into other disciplines of medical and public health research.

## Supporting information

S1 Fig Diagram of the Bayesian HNB model. Hierarchical diagram of the relationships between hyper-parameters and parameters involved in the EM-IWLS algorithm for fitting the Bayesian HNB model.
(PDF)

S2 Fig Comparison of the Bayesian HNB model and the classical NB model taking into account the simulation of human microbiome count data with 1 covariate. **(A)** Type I error and **(B)** power for fitting a single covariate using the Bayesian HNB model compared to the three competing modeling over combinations of sample size and effect size. The Bayesian HNB model using nine prior scales is represented by an increasing sequential palette of yellows, oranges, and reds while *MASS* is represented by green, *edgeR* is represented by blue, and *DESeq2* is represented by purple.
(PDF)

S3 Fig Comparison of the Bayesian HNB model and three competing models taking into account the simulation of human microbiome count data with 100 covariates. Jointly fitting 100 covariates using the Bayesian HNB model (yellows, oranges, and reds) compared to *MASS* (green), *edgeR* (blue), and *DESeq2* (purple)stratified by sample size (right) and correlation (top). Increasing prior scale is represented by an increasing sequential palette detailed in the legend. Each row of the individual panels represents 1 of the 50 covariate with the gray dashed lines associated with *β*_15_∈(0.01,0.15), *β*_30_∈(0.20,0.35), and *β*_45_∈(0.40,0.55) corresponding to the three simulated covariates assigned to have non-zero effects from small, moderate, and large range, respectively. Frequency along said gray dashed lines represents power. All additional effects were simulated to be the value of 0 such that frequency represents type I error.
(PDF)

S4 Fig Comparison of the Bayesian HNB model and three competing models taking into account the simulation of human microbiome count data with 200 covariates. Jointly fitting 200 covariates using the Bayesian HNB model (yellows, oranges, and reds) compared to *MASS* (green), *edgeR* (blue), and *DESeq2* (purple) stratified by sample size (right) and correlation (top). Increasing prior scale is represented by an increasing sequential palette detailed in the legend. Each row of the individual panels represents 1 of the 50 covariates with the gray dashed lines associated with *β*_15_∈(0.01,0.15), *β*_30_∈(0.20,0.35), and *β*_45_∈(0.40,0.55) corresponding to the three simulated covariates assigned to have non-zero effects from small, moderate, and large range, respectively. Frequency along said gray dashed lines represents power. All additional effects were simulated to be 0 such that frequency represents type I error.
(PDF)

S5 Fig Computation time. Mean ±2 standard error computation time for the Bayesian HNB model (orange) compared to *MASS* (green), *edgeR* (blue), and *DESeq2* (purple) stratified by sample size (top) and reported in minutes.
(PDF)

S6 Fig Convergence. Mean ±2 standard error convergent iterations for the Bayesian HNB model (orange) compared to *MASS* (green), *edgeR* (blue), and *DESeq2* (purple) stratified by sample size (right) and number of covariates (top).
(PDF)

S7 Fig Overlap of differentially abundant OTUs for application of the Bayesian HNB model and three competing models to reduced and full AGP subsets. Venn diagrams displaying the numbers of differentially abundant OTUs identified by the Bayesian HNB model utilizing (A) reduced and (B) full AGP subsets compared to the classical NB model utilizing (C) reduced and (D) full AGP subsets.
(PDF)

S1 Table Description of reduced AGP subsets. Summary statistics for the host characteristics of the reduced AGP subsets consisting of 4 demographic attributes, 32 dietary behaviors, and 6 systemic practices stratified across IBD and IBS classifications.
(XLSX)

S2 Table Description of full AGP subsets. Summary statistics for the host characteristics of the full AGP subsets consisting of 4 demographic attributes, 32 dietary behaviors, and 6 systemic practices stratified across IBD and IBS classifications.
(XLSX)

S3 Table Comparison of AIC statistics for application of the Bayesian HNB model and the classical NB model to reduced and full AGP subsets. AIC statistics for the differential abundance analysis of IBD and IBS in respect to reduced and full AGP subsets analyzed using the Bayesian HNB and classical NB models. Degrees of freedom adjusted to the effective number of parameters prior to calculation of the AIC statistic for each application of the Bayesian HNB model. Minimized AIC suggests stronger evidence for model selection given an optimal prior scale supporting inclusion in S4 Table.
(XLSX)

S4 Table Comparison of coefficient estimates and p-values for application of the Bayesian HNB model and the three competing models to reduced and full AGP subsets. Coefficient estimates and p-value for the differential abundance analysis of IBD and IBS in respect to reduced and full AGP subsets analyzed using the Bayesian HNB model and the classical NB model. Prior scale for Bayesian HNB model selected based on minimized adjusted AIC as shown in S3 Table. Only those OTUs with at least one p-value below the significance level of 0.10 are included.
(XLSX)
