# Supplementary figures and images for: Bayesian hierarchical negative binomial models for multivariable analyses with applications to human microbiome count data
Source: PLoS One. 2019 Aug 22;14(8):e0220961. doi: 10.1371/journal.pone.0220961 (PMC6706006; doi:10.1371/journal.pone.0220961)

Initialize Model Parameters:

E-Step:

M-Step:

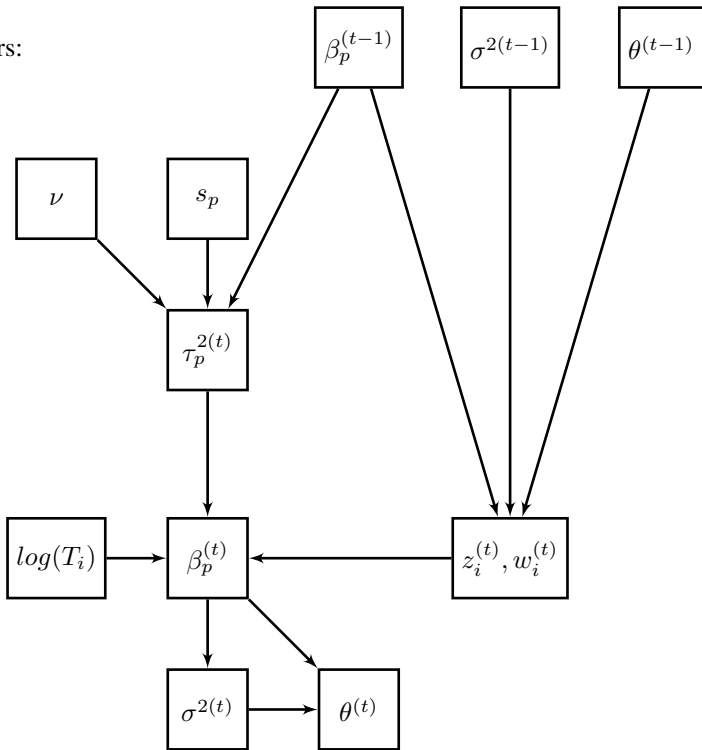

Supplement: S1 Fig — Hierarchical diagram of the relationships between hyper-parameters and parameters involved in the EM-IWLS algorithm for fitting the Bayesian HNB model. (PDF) [file pone.0220961.s001.pdf]

**A**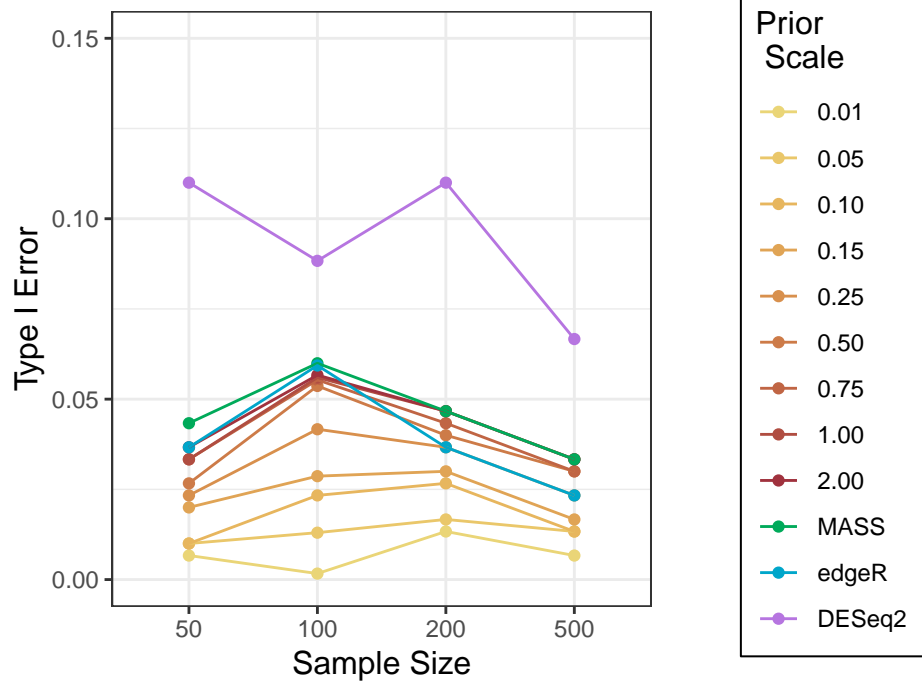**B**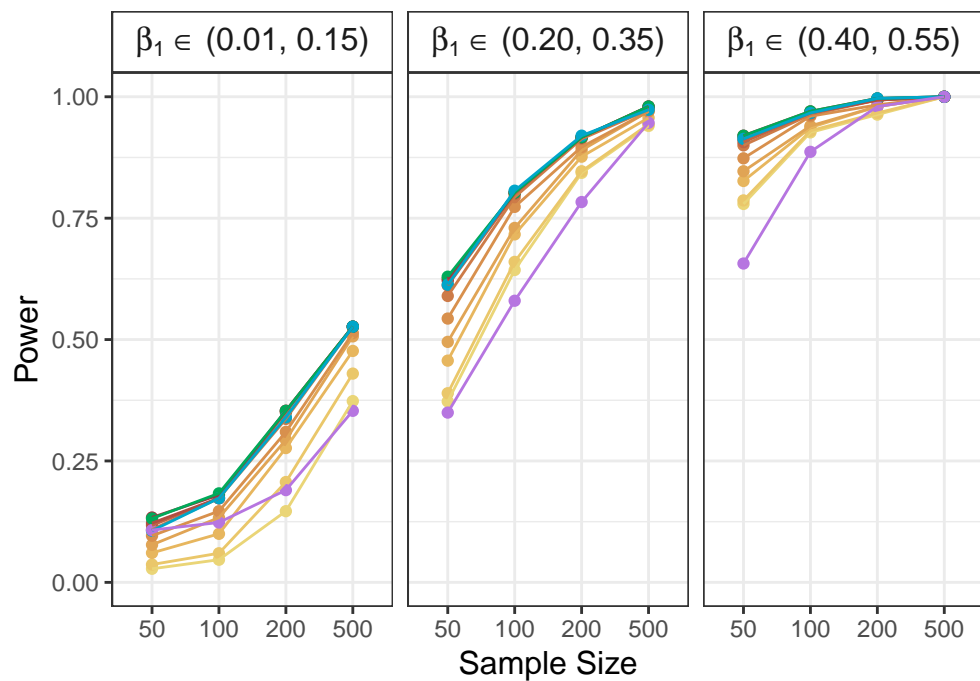

Supplement: S2 Fig — (A) Type I error and (B) power for fitting a single covariate using the Bayesian HNB model compared to the three competing modeling over combinations of sample size and effect size. The Bayesian HNB model using nine prior scales is represented by an increasing sequential palette of yellows, oranges, and reds while MASS is represented by green, edgeR is represented by blue, and DESeq2 is represented by purple. (PDF) [file pone.0220961.s002.pdf]

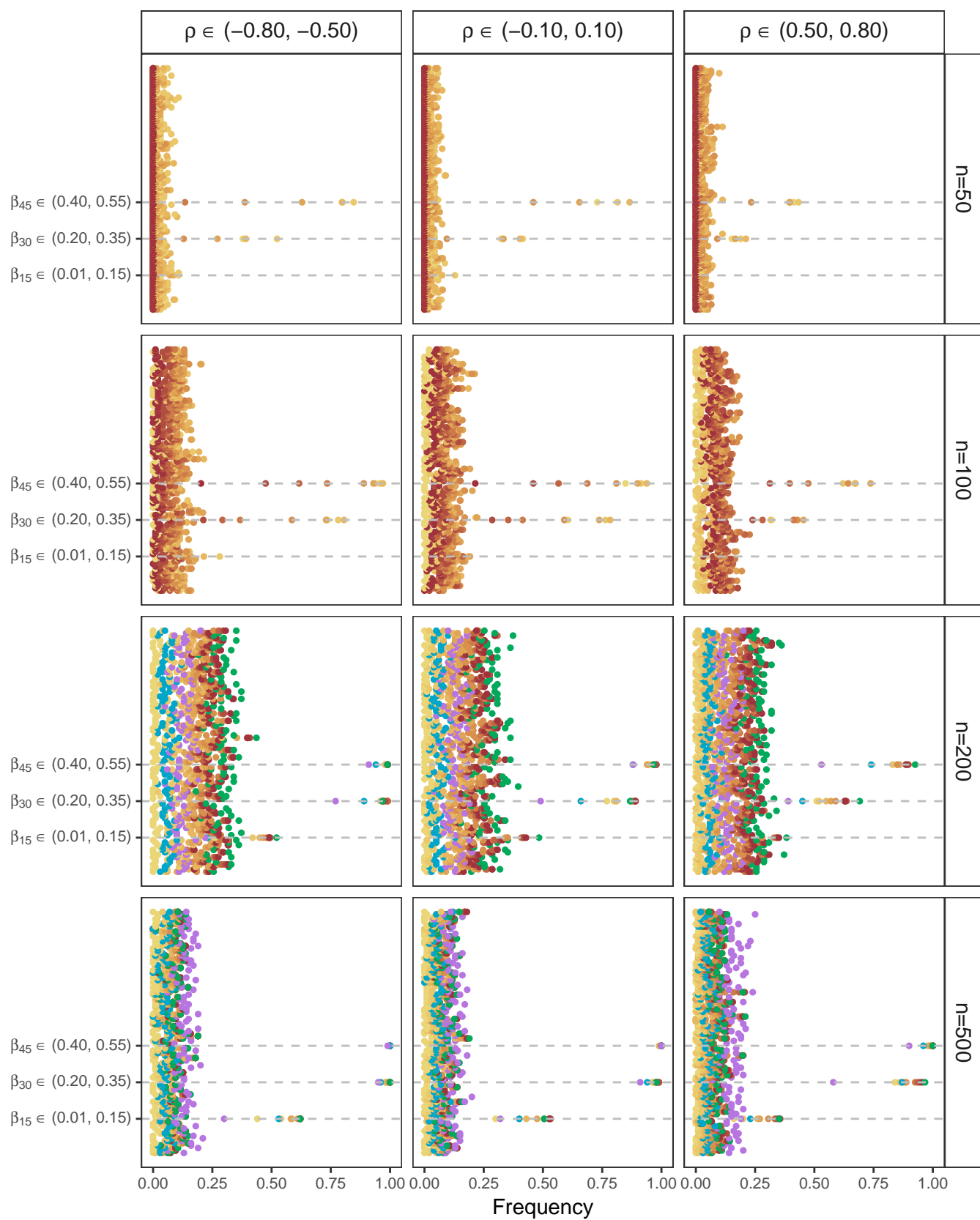

Supplement: S3 Fig — Jointly fitting 100 covariates using the Bayesian HNB model (yellows, oranges, and reds) compared to MASS (green), edgeR (blue), and DESeq2 (purple)stratified by sample size (right) and correlation (top). Increasing prior scale is represented by an increasing sequential palette detailed in the legend. Each row of the individual panels represents 1 of the 50 covariate with the gray dashed lines associated with β15∈(0.01,0.15), β30∈(0.20,0.35), and β45∈(0.40,0.55) corresponding to the three simulated covariates assigned to have non-zero effects from small, moderate, and large range, respectively. Frequency along said gray dashed lines represents power. All additional effects were simulated to be the value of 0 such that frequency represents type I error. (PDF) [file pone.0220961.s003.pdf]

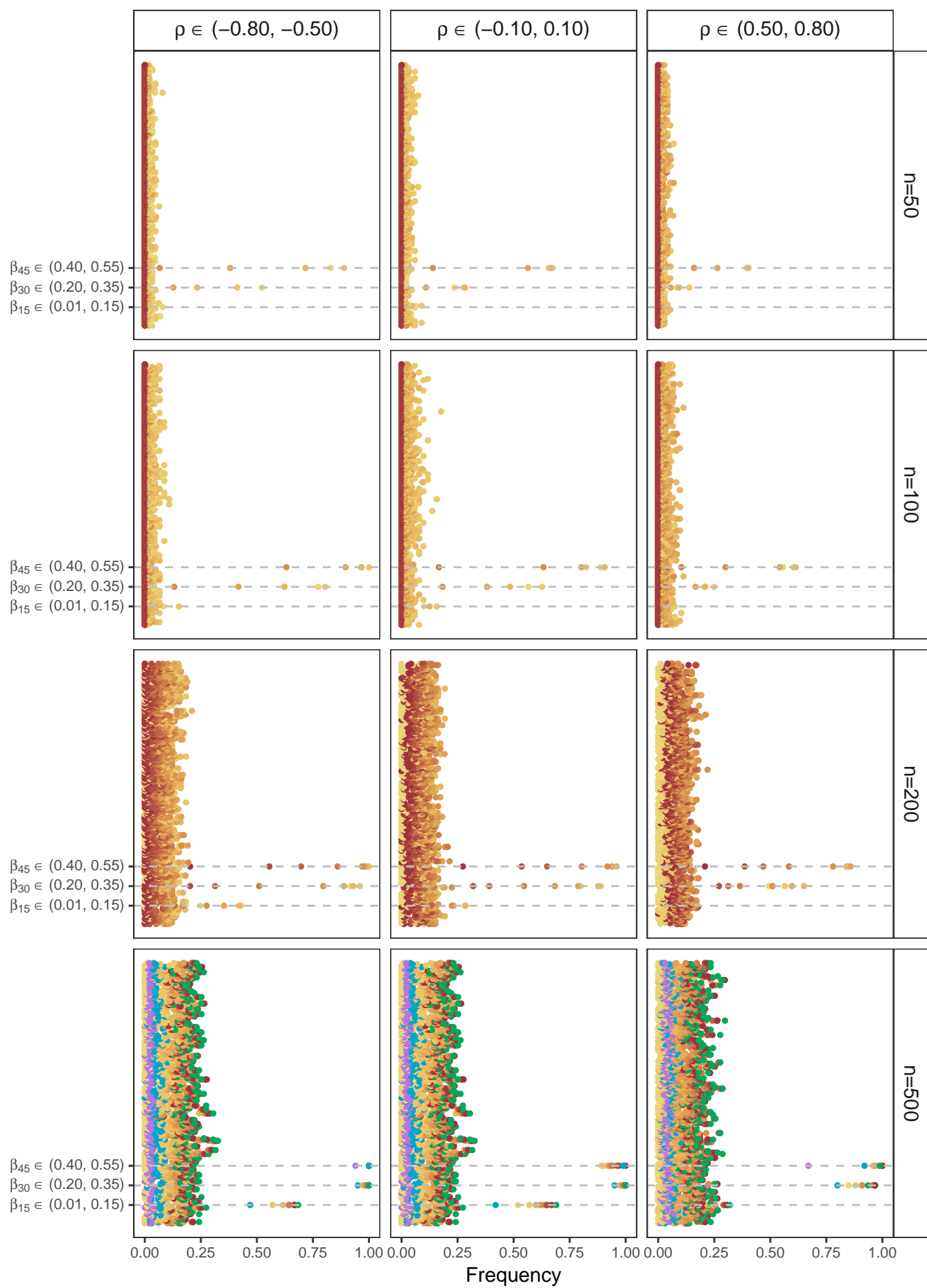

Supplement: S4 Fig — Jointly fitting 200 covariates using the Bayesian HNB model (yellows, oranges, and reds) compared to MASS (green), edgeR (blue), and DESeq2 (purple) stratified by sample size (right) and correlation (top). Increasing prior scale is represented by an increasing sequential palette detailed in the legend. Each row of the individual panels represents 1 of the 50 covariates with the gray dashed lines associated with β15∈(0.01,0.15), β30∈(0.20,0.35), and β45∈(0.40,0.55) corresponding to the three simulated covariates assigned to have non-zero effects from small, moderate, and large range, respectively. Frequency along said gray dashed lines represents power. All additional effects were simulated to be 0 such that frequency represents type I error. (PDF) [file pone.0220961.s004.pdf]

Computation Time

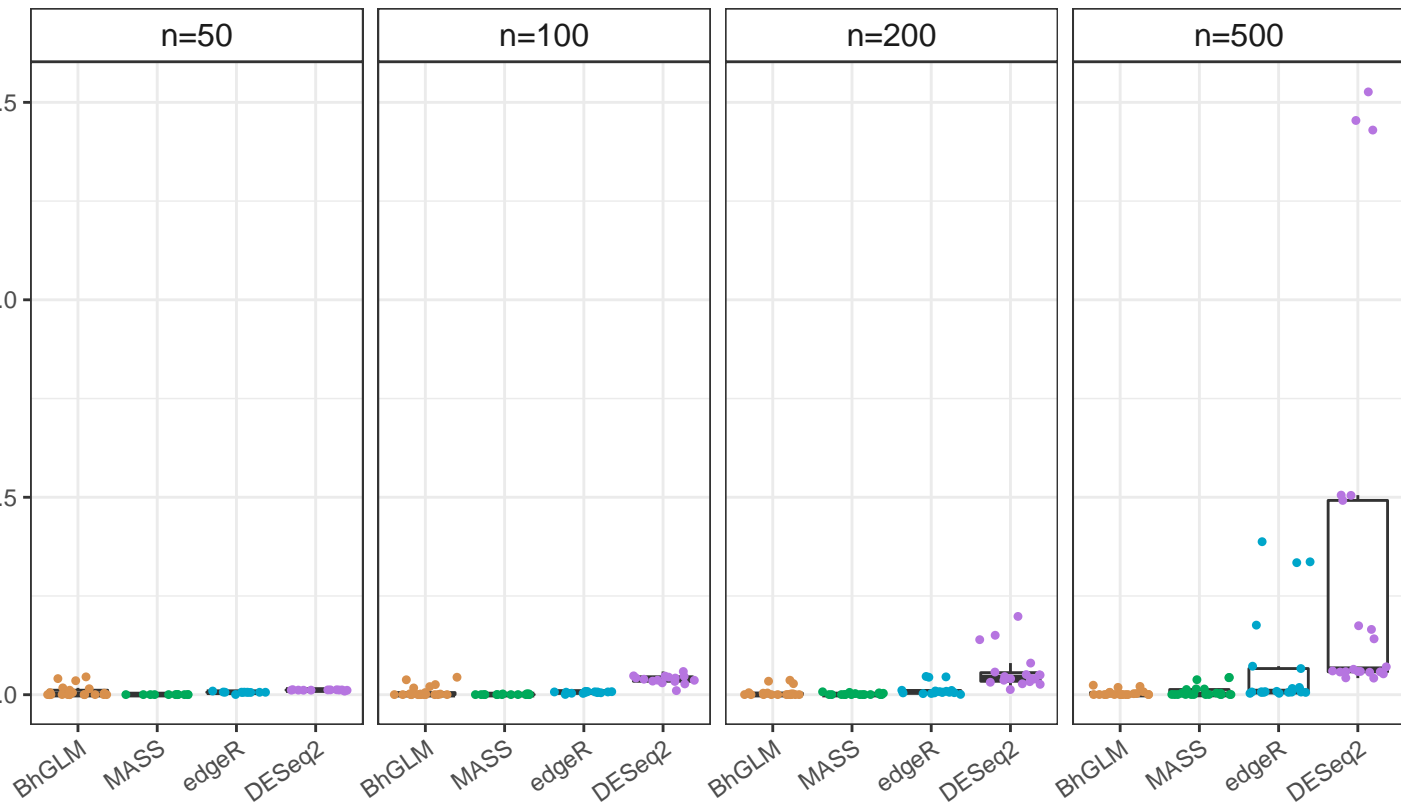

Supplement: S5 Fig — Mean ±2 standard error computation time for the Bayesian HNB model (orange) compared to MASS (green), edgeR (blue), and DESeq2 (purple) stratified by sample size (top) and reported in minutes. (PDF) [file pone.0220961.s005.pdf]

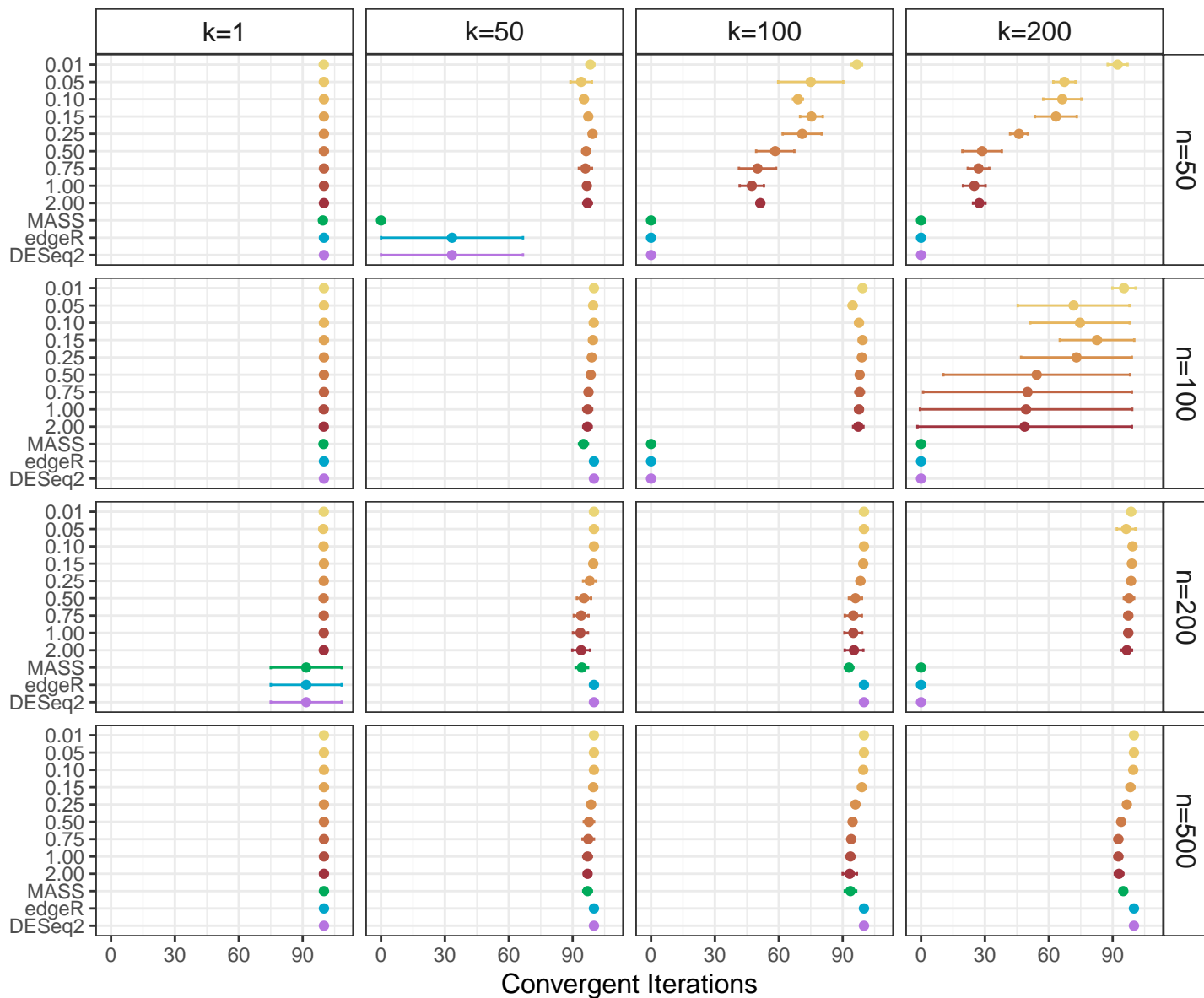

Supplement: S6 Fig — Mean ±2 standard error convergent iterations for the Bayesian HNB model (orange) compared to MASS (green), edgeR (blue), and DESeq2 (purple) stratified by sample size (right) and number of covariates (top). (PDF) [file pone.0220961.s006.pdf]

IBD

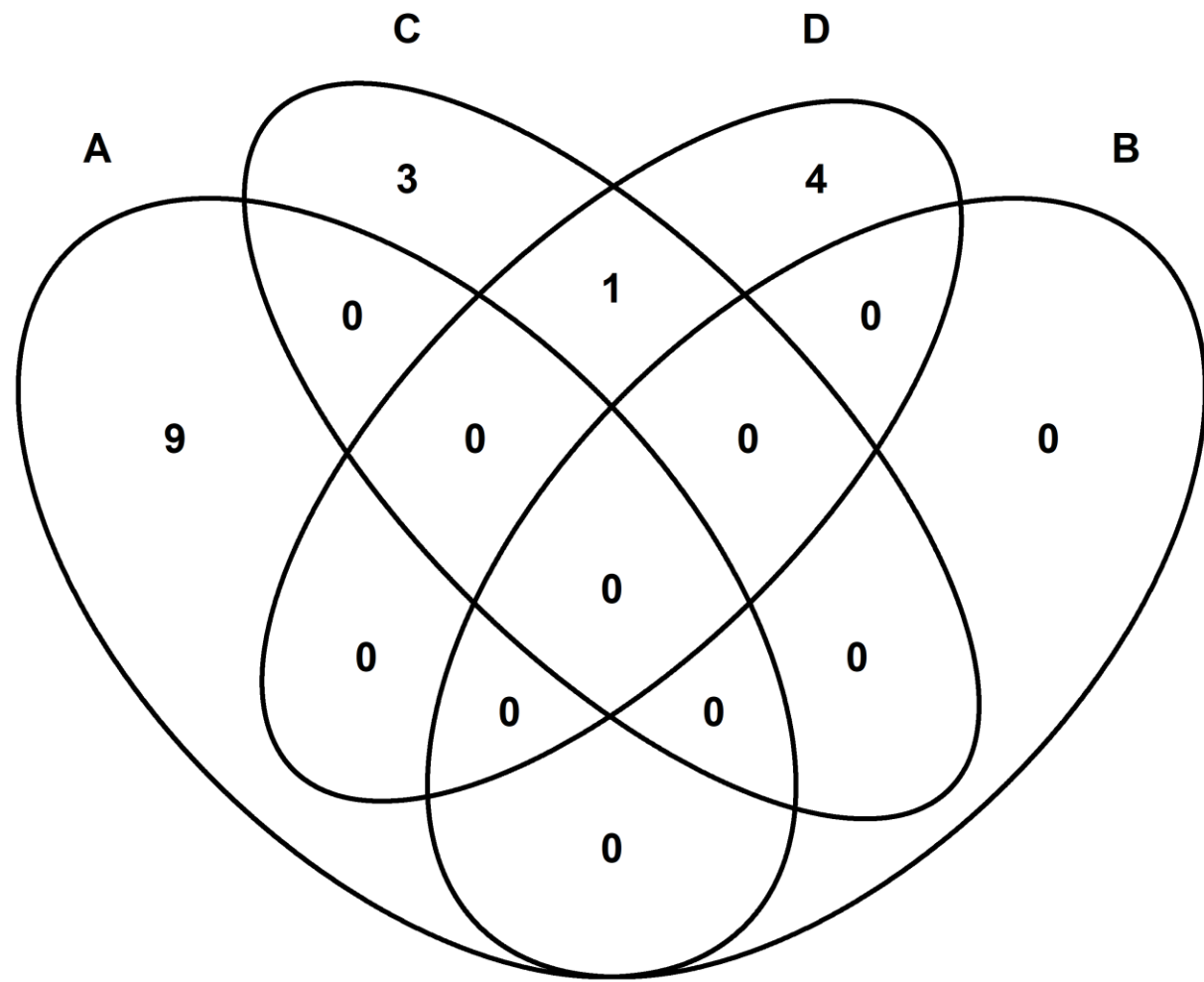

IBS

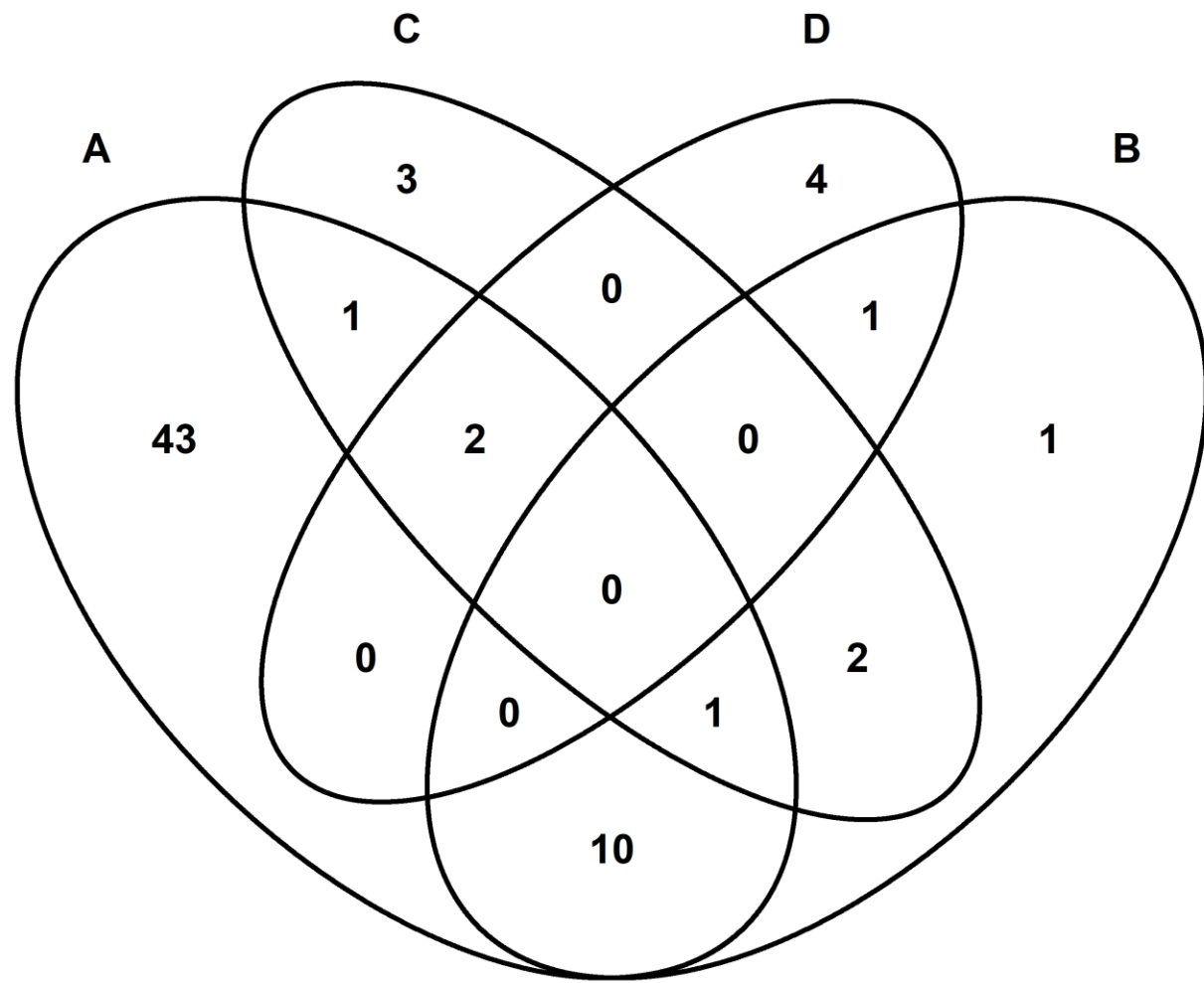

Supplement: S7 Fig — Venn diagrams displaying the numbers of differentially abundant OTUs identified by the Bayesian HNB model utilizing (A) reduced and (B) full AGP subsets compared to the classical NB model utilizing (C) reduced and (D) full AGP subsets. (PDF) [file pone.0220961.s007.pdf]
